# The burden of delayed diagnosis in Hirschsprung disease: insights from a Paediatric Colorectal Centre in South Africa

**DOI:** 10.1186/s12887-025-05898-w

**Published:** 2025-07-10

**Authors:** Emanuele Trovalusci, Tebogo Thelele, Paola Midrio, Catterina Bebington, Chris Westgarth-Taylor, Giulia Brisighelli

**Affiliations:** 1 Paediatric Surgery Unit, Department of Women’s and Children’s Health, University of Padua, Padua, Italy; 2 Department of Paediatric Surgery, Johannesburg Paediatric Colorectal Clinic - Chris Hani Baragwanath Academic Hospital, University of the Witwatersrand, Johannesburg, South Africa; 3 Department of Paediatric Surgery - Ca’ Foncello Hospital, Treviso, Italy; 4 Pediatric Colorectal and Urogenital Program, Christus Children’s Hospital, Baylor College of Medicine, San Antonio, TX USA

**Keywords:** Delayed diagnosis, Hirschsprung disease, Africa, Paediatric colorectal

## Abstract

**Background:**

Delayed diagnosis of Hirschsprung disease (HD), defined as diagnosis after 12 months of age, is usually uncommon and, according to some authors, associated with a worse outcome. We aim to report our experience in a tertiary hospital in a Low-and-Middle-Income Country (LMIC).

**Methods:**

A retrospective analysis was conducted using the RedCap^®^ Database of the Johannesburg Paediatric Colorectal Clinic, which includes data from all HD patients since 2017. Patients who had pull-through surgery in other hospitals were excluded. The remaining patients were divided into delayed and non-delayed. Demographic and clinical data, including the number and type of surgeries, complications, and functional outcomes, were collected and compared between the two groups using the Mann-Whitney U and Fisher’s Exact Test.

**Results:**

Of 80 patients with HD, 26 (32.5%) had a delayed diagnosis, with a median age of 3.14 (1–15) years. Of these, 5 patients were excluded from further analyses because they underwent a pull-through elsewhere. Patients with a delayed diagnosis underwent a median of 4 surgical procedures, compared to 3 in those diagnosed earlier (*p* = 0.013), but 97.5% of them required surgical diagnostic biopsies, compared to 23.8% in the non-delayed group (*p* < 0.0001).

No differences have been noticed in terms of the type of pull-through performed, number of complications and additional procedures required. Incontinence, constipation, and enterocolitis episodes were similar in both groups.

**Conclusions:**

Delayed diagnosis of HD can be a burden for the healthcare system, as these patients usually require more surgical procedures compared to non-delayed patients, but even in a LMIC context, no significant differences have been seen in terms of complications and post-surgical outcomes, as well as bowel function.

## Introduction

Hirschsprung’s disease (HD) is a rare cause of neonatal intestinal obstruction, with an incidence estimated to be 1 in 5,000 live births but with a range varying from 1 in 2,000 to 1 in 12,000 live births [1]. It is caused by the absence of ganglion cells in the distal colonic segment, which prevents the propagation of peristaltic waves and determines a narrowing of the affected bowel, plus a dilation of the proximal one secondary to stasis of the stools. Males are more frequently affected (4:1 ratio) [2], but this preponderance is less evident in the long-segment types (1:1–2:1) [3].

HD is typically diagnosed within the first month of life, often suspected when there is delayed passage of meconium (> 48 h), abdominal distension, and vomiting. However, in some cases, both short and long segment forms of HD may present with more subtle symptoms and lead to misdiagnosis or delayed diagnosis [4].

Current literature lacks a standardised definition for late diagnosis of HD, with age cut-offs varying widely from one week after birth to three years of age [5].

Although it was initially believed that late detection of HD was associated with an increased risk of complications [5], more recent studies have not observed a worse prognosis in cases of late-diagnosed HD [6, 7]. Unfortunately, data about this topic is still scarce, and most of these studies have been conducted in high-income countries.

This report aims to present the experience in managing the delayed presentation of HD cases in the Johannesburg Paediatric Colorectal Clinic (JPCC) at Chris Hani Baragwanath Academic Hospital (CHBAH), based in Johannesburg, South Africa. The primary aim of this study is to observe if there is a higher rate of morbidity, mortality, and number of complications in patients with a delayed diagnosis. The secondary aim is to assess the functional outcomes in terms of bowel motility, enterocolitis episodes, and quality of life.

## Materials and methods

This study is a retrospective analysis of the medical records of all patients with HD treated and followed up at the JPCC at CHBAH between 2017 and 2024. All the data utilised for this analysis was drawn from the JPCC REDCap^®^ Database.

For this study, we examined all patients with HD and categorized them into two groups: those who received a delayed diagnosis and those who received a timely diagnosis. A delayed diagnosis was defined as HD diagnosed after one year of age.

Data from both groups were analysed individually and compared to assess differences in presentation, management, and outcomes.

Inclusion criteria were: confirmed histological diagnosis of HD, sufficient data on presenting symptoms and surgical history, and parental consent to analyse medical data.

Patients who were followed up at JPCC but underwent pull-through surgery at other institutions were excluded to minimize inconsistencies in diagnostic and surgical data, as well as variations in surgical techniques compared to those used at our Centre.

Data analysed included demographic data (sex, age at diagnosis, age at pull-through surgery, hospital of initial management), medical variables (length of the aganglionic segment, associated comorbidities, diagnostic procedures, and surgical procedures – stoma, pull-through surgery, other procedures post-pull-through), and bowel outcomes (episodes of HAEC, faecal incontinence, and overall bowel function, assessed with the Rintala score [8]). Mortality data (age and cause) was also recorded. Categorical data are reported as frequencies and percentages, while continuous variables are described using medians and ranges for abnormally distributed variables.

Comparison between the case and control cohorts has been evaluated using the Mann-Whitney U-Test for continuous variables and Fisher’s Exact Test for categorical variables.

A *p*-value threshold of < 0.05 was set to determine statistical significance.

Ethics approval for this study was granted under protocol M190508, ensuring that the research meets ethical standards for patient confidentiality, data integrity, and responsible reporting. 

## Results

A total of 80 patients with Hirschsprung disease (HD) have been followed up at JPCC in the period between 2017 and 2024. The median follow-up period was 4.2 years (range: 3 months – 6.9 years).

Of these, 26 (32.5%) received a delayed diagnosis, being confirmed at histology only after their first year of life. This delayed group had a median age at diagnosis of 3.14 years (range: 1–15 years). The non-delayed group consisted of 54 patients and had a median age at diagnosis of 61 days (range: 3-307 days).

Sixty-six patients out of 80 (82.5%) were males, with 22/26 (84.6%) in the delayed diagnosis group.

The information on the specific type of HD was available for 22/26 (84.6%) patients among the delayed diagnosis group and for 40/54 (74.1%) patients in the non-delayed one; no statistically significant difference has been noted in the prevalence of HD subtypes amongst the two groups (*p* = 0.35). The subtypes are reported in Table 1.

**Table 1:** Demographic data, age at diagnosis and type of HD in delayed and non-delayed HD patients

	Delayed	Non-delayed	*p*-value
Number of patients	26/80 (32.5%)	54/80 (67.5%)	
Age at diagnosis	3.14 years (1–15 years)	61 days (3-307 days)	0.01
Male sex	84.6%	81.5%	0.99
Age at pull-through	3 years (1.2–15.3 years)	0.9 years (0.1–2.1 years)	0.01
Type of HD			
Known	22/26 (84.6%)	40/54 (74.1%)	0.29
Recto-sigmoid	16/22 (72.7%)	25/40 (62.5%)	0.35
Long segment	4/22 (18.2%)	5/40 (12.5%)	
Total colonic aganglionosis	2/22 (9.1%)	10/40 (25%)	

No familial history was noted in the delayed group, while only one patient in the control group had two siblings diagnosed with HD. No significant differences were observed in terms of associated comorbidities and syndromes.

Out of the 80 patients followed-up at JPCC, 69 (86.2%) had a pull-through performed at our institution, and 11 at a different institution (5/26 patients with delayed HD − 19.2%; 6/54 with non-delayed HD − 11.1%); an additional 3 patients in the non-delayed group presented incomplete medical records, leaving a total of 21 (80.8%) patients in the delayed group and 45 (83.3%) in the non-delayed group eligible candidates for the study.

### Diagnostic procedures

**Fig. 1:**
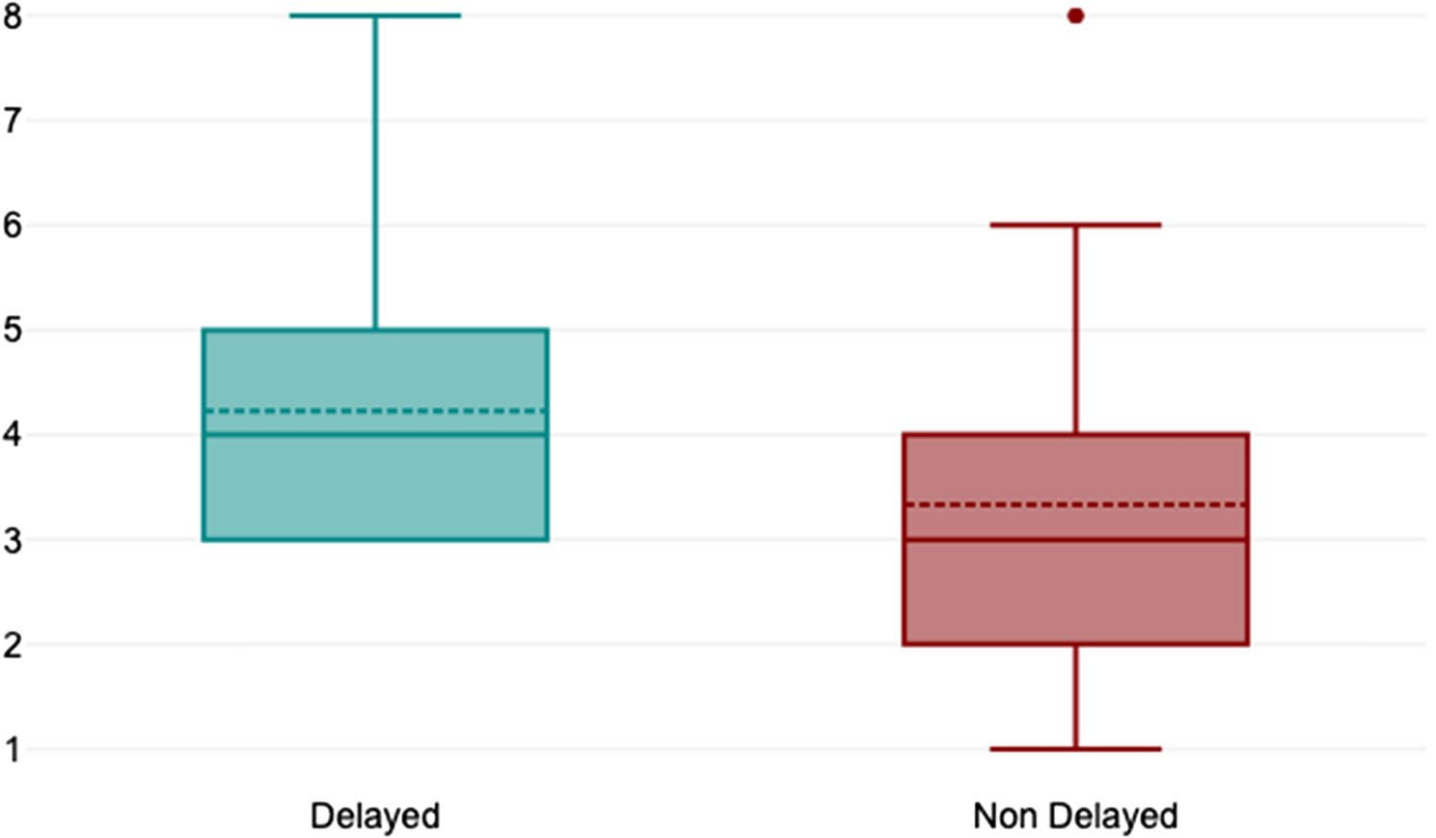
Number of surgical procedures in delayed and non-delayed HD patient. *P* = 0.0013

**Fig. 2:**
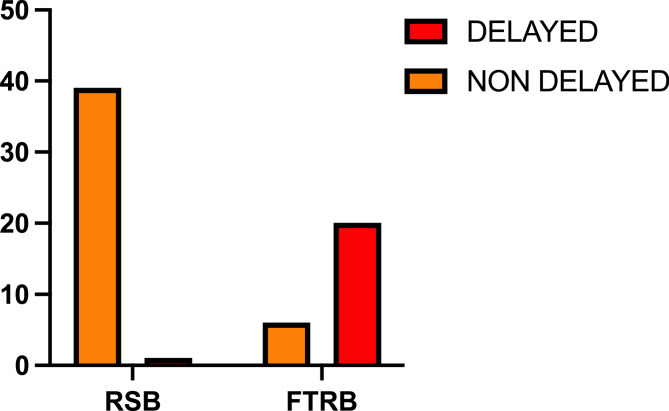
Number and type of biopsies in delayed and non-delayed HD patients. *P* < 0.0001

Patients with delayed diagnosis of Hirschsprung disease (HD) underwent a median of 4 surgical procedures (range: 3–8), compared to 3 in those non-delayed (range 1–8), with an exact *p*-value = 0.013 (Fig. 1). Twenty out of 21 patients (95.2%) in the delayed group required surgical diagnostic biopsies, compared to 6/45 (13.3%) in the non-delayed group (*p* = 0.01) (Fig. 2).

### Stoma and pull-through procedures

Abdominal Swenson technique was the only pull-through method performed at our institution. In 61/66 cases (92.4%) a staged procedure was performed (stoma fashioning followed by pull-through of stoma) (Table 2).

**Table 2:** Summary of pull-through and post-pull-through procedures in delayed and non-delayed HD patients

	Delayed	Non-delayed	*p*
**Pull-through**	21/21 (100%)	43/45 (96.2%)	0.99
Primary	1/21 (4.8%)	4/45 (8.9%)	0.99
Staged	20/21 (95.2%)	41/45 (91.1%)	0.99
Re-do	0/21 (0%)	1/45 (2.2%)	0.99
**Additional procedures**			
EUA	10/21 (47.6%) – *20 procedures*	20/45 (44.4%) – *36 procedures*	0.99
Botulinum injection	4/21 (19.1%) – *6 procedures*	10/45 (22.2%) – *16 procedures*	0.99
Repeated FTRB	2/21 (9.5%) – *2 procedures*	9/45 (20.0%) – *12 procedures*	0.48
**Management of complications**			
Adhesiolysis	1/21 (4.8%)	3/45 (6.6%)	0.95
Pelvic collection drainage	1/21 (4.8%)	2/45 (4.4%)	0.99

Stoma revision secondary to prolapse was required in 1/22 (4.5%) patients in the delayed group and 2/45 (4.4%) in the non-delayed group. Additionally in the delayed group, 1/22 (4.5%) patients required a repeat biopsy of the colostomy before the pull-through to confirm the presence of ganglion cells at that level.

### Post-pull-through surgeries

Table 2 summarizes the surgeries performed post pull-through in the two groups of patients.

No significant difference was noted in the number of redo procedures between the delayed and non-delayed group, with only one patient in the non-delayed group requiring a redo procedure due to stricture of the anastomosis that was refractory to dilatations.

Pelvic collections secondary to anastomotic leakage required surgical drainage and a covering ileostomy in 1 patient from the delayed group and in 2 patients from the non-delayed group, with one of the non-delayed patients also developing a recto-vaginal fistula that required repair (4.8% vs. 6.6%, *p* = 0.32).

No significant differences were found in terms of bowel obstruction secondary to adhesions. Exploratory laparotomy and adhesiolysis were required in 1/21 of the delayed HD group and 3/45 patients in the non-delayed group (4.8% vs. 6.7%, *p* > 0.99).

Additionally, no difference was observed between the delayed and non-delayed diagnosis groups in the frequency of post-pull-through procedures, such as examination under anaesthesia to check the patency of the anastomosis, botulinum injections, or repeated full-thickness rectal biopsies (FTRB) to exclude the pull-through of the transition zone/aganglionic part of the colon, which was always excluded in our cohort of patients.

### Functional outcomes

**Fig. 3:**
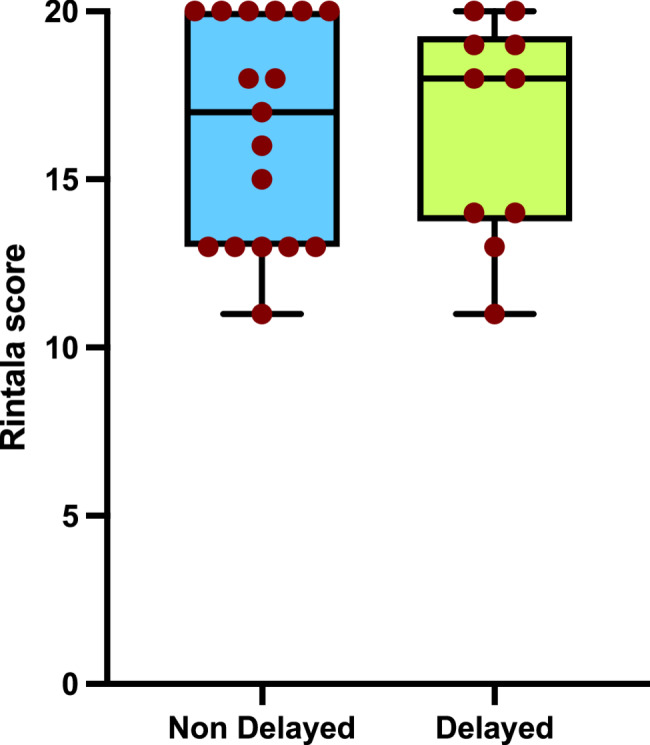
Rintala score comparison between delayed and non-delayed HD patients. *P* = 0.92

Bowel function, assessed using the Rintala score after 3 years of age, was evaluated in 10/21 (47.6%) patients in the delayed group and 17/45 (37.8%) in the non-delayed group. The median scores were 18 and 17 for delayed and non-delayed groups, respectively, with a range between 11 and 20 in both groups (*p* = 0.92) (Fig. 3). Bowel function is typically assessed after the age of 3. The median age at assessment was 6.2 years (range 3.5–13.6) for the delayed group and 4.2 years (range 1.4–12.6) for the non-delayed group (*p* = 0.04).

In particular, 2/10 (20%) patients in the delayed group reported soiling (2 once a month or less, 1 once a week or less), compared to 6/17 (35.3%) in the non-delayed group (3 once a month or less, 3 once a week or less), with no statistical significance noted (*p* = 0.66). No differences were also noted in terms of constipation requiring occasional or prolonged use of laxatives (3/10 vs. 6/17) (30% vs. 35.3%) (*p* = 0.99).

Regarding true incontinence, no significant differences were observed between the two groups. Specifically, 2/21 patients in the delayed group and 3/45 in the non-delayed group presented with incontinence (9.5% vs. 6.7%, *p* = 0.65). None of the patients who had a primary pull-through developed faecal incontinence. All cases of incontinence showed similar anatomical outcomes, with a disruption of the dentate line when examined under anaesthesia.

In terms of enterocolitis incidence, both pre- and post-operatively: 3/21 patients in the delayed group experienced at least one episode of HAEC during their life, as did 6/45 patients in the non-delayed group (14.3% vs. 13.3%, *p* = 0.99).

In relation to mortality, we observed 3 deaths in the non-delayed group and 1 in the delayed group. Information regarding the causes is available for only 2/3 patients (both in non-delayed group): one died due to severe wasting after being lost to follow-up before undergoing stoma/pull-through, and the other succumbed to septic shock secondary to intraperitoneal faecal contamination following a dehiscence at the site of a full-thickness colonic biopsy performed during a laparotomy for ileostomy fashioning.

## Discussion

Late diagnosis of HD is relatively uncommon in high-income countries, with only a small proportion of cases presenting in childhood, adolescence, or even adulthood (7.7–18.4%) [5, 6]. In contrast, delayed diagnosis is more frequent in low-income countries due to barriers to healthcare access, including long distances to hospitals, unrecognised symptoms by midwives and parents, a shortage of trained healthcare workers, and persistent reliance on traditional healers due to a lack of awareness [9]. As a result, the prevalence of delayed HD diagnosis is higher in LMICs, despite data in the literature about the real prevalence still being scarce, with a reported prevalence between 27% and 57.7%. This aligns with the prevalence observed in our cohort of patients (33%) [9–11].

Cultural beliefs and traditional practices further contribute to delays in seeking medical care. In South Africa, for instance, around 80% of black South Africans consult a traditional healer before attending a hospital or clinic. These practitioners – including diviners (Sangomas), herbalists, spiritual healers, or traditional birth attendants – play a significant role in healthcare decision making [12]. The administration of enemas is a common practice in traditional medicine within the Zulu South African community. A local study found that 53% of infants received multiple weekly enemas during the first six weeks of life, both due to a false perception of constipation and to protect the child by purging harmful evil influences [13]. Unfortunately, this practice is not only potentially ineffective but could also delay a necessary referral to the hospital in the case of an underlying medical condition or cause harm to the child.

In our setting, delayed diagnosis is often due to a combination of limited awareness among patients and healthcare workers, and the widespread use of traditional enemas. These factors complicate timely recognition of HD symptoms and are exacerbated by difficulties in obtaining accurate medical histories because of incomplete medical records and parental reluctance to disclose traditional medicine use.

In addition to this phenomenon of delayed patient referral to hospitals, recent studies have postulated that delayed cases exhibit less pronounced and typical symptoms of HD; it has been observed that patients with delayed diagnosis show a lower incidence of delayed meconium passage compared to typical HD cases (35% vs. 64% [6, 14]). This suggests that these patients may exhibit milder symptoms, which can be easily overlooked during the neonatal period, frequently resulting in a diagnosis and treatment plan focused on functional constipation rather than HD. Unfortunately, it was not possible to analyse retrospectively the presenting symptoms in our series.

Pini Prato et al. observed in their series a high incidence (20%) of associated anomalies, such as Down Syndrome, congenital heart disease, and congenital central hypoventilation syndrome, among patients with delayed HD diagnosis [14]. The presence of such significant issues can divert clinical focus from gastrointestinal symptoms, especially in milder cases, contributing to a higher risk of delayed diagnosis. This observation is likely accurate, though we didn’t observe a comparable frequency of these conditions in our population of delayed HD, with no cases of Down Syndrome or congenital central hypoventilation syndrome and only mild forms of cardiac anomalies. However, we do not routinely screen for cardiac anomalies in our clinical practice, which could account for this difference.

Literature also reports that long-segment HD or total colonic HD can lead to a delayed diagnosis, as meconium passage may appear normal, despite typically more severe symptoms [15–17]. In our cohort, however, these forms were observed in 18.2% and 9.1% of patients, respectively, indicating that the classic form of HD remains predominant also in our setting and that these variants should not be considered risk factors for delayed diagnosis, considering that no statistical significance has been noted with the non-delayed group.

This aligns with findings from a large systematic review by Doodnath and Puri, which included 490 HD patients diagnosed between the ages of 11 and 79 years with an incidence of the recto-sigmoid type of 78.9% [18].

At our institution, we typically perform an end stoma and a staged repair in all HD patients. This approach is largely driven by the high risk of sepsis and anastomotic dehiscence, which is further compounded by an increased prevalence of poor nutritional status and HIV exposure among South African children [19]. Additionally, it ensures a safer discharge without the need for bowel irrigations. In contrast, Ostertag-Hill reported successful management of the challenges posed by a massively dilated colon by implementing at-home bowel decompression with irrigations and resecting an extended portion of the colon to ensure an anastomosis in a non-dilated segment. This approach allowed for a primary pull-through in 85% of cases, with only 2 patients requiring a covering ostomy [7]. Similarly, Pini Prato reported that 20% of patients required a stoma—10% before the pull-through procedure and another 10% as a covering stoma during the pull-through [6, 20]. Both these studies originate from HIC where healthcare access and patient follow-up are more reliable. We do not believe that this approach could be feasible in our setting due to the risk of discharging a child on irrigations. If not performed correctly, irrigations could lead to HAEC, which can be fatal – particularly in a population already facing substantial barriers in healthcare access.

We did not observe the need for a permanent stoma in any of our delayed diagnosis HD patients, nor do we routinely perform a covering ileostomy in all delayed HD patients undergoing a pull-through procedure. In our series, none of our delayed diagnosis patients required a permanent stoma, in contrast with what was reported by Tan [5]. Therefore, similarly to what was observed by Pini Prato, the assumption that misdiagnosis and delay of referral leads to worse post-operative outcomes is not supported by our findings [6].

To our knowledge, no previous studies have reported an increased number of required surgical procedures for patients with a delayed diagnosis of HD. However, in our series, this higher rate of surgical interventions can be attributed to the need for surgical rectal biopsies under anaesthesia. Since rectal suction biopsies are often unreliable in patients over one year of age, a surgical biopsy becomes necessary to confirm the diagnosis [20]. This highlights both the procedural and economic impact of delayed diagnosis on the healthcare system. Therefore, given the high prevalence of delayed diagnoses in our setting, maintaining a low threshold for recommending surgical biopsy in patients presenting with persistent constipation is crucial.

While initial studies suggested a higher rate of post-surgical complications in patients with delayed diagnosis HD, particularly concerning anastomotic leaks [21], more recent research has not supported these findings [7, 18]. In our cohort, we observed no significant differences in the rates of anastomotic leaks or pelvic abscesses. Additionally, we found no variation in other early- or mid-term complications, such as bowel obstruction due to adhesions or the need for stoma surgeries, either for stoma-related issues or for creating covering stomas.

We also did not observe differences between groups in the frequency of procedures such as examinations under anaesthesia to assess anastomotic patency, botulinum injections, or repeat full-thickness rectal biopsies (FTRBs) to rule out retained aganglionosis or transition zone pull-through.

Ostertag-Hill et al. reported a faecal incontinence rate of 16.7% (consistent with our findings) and a constipation rate of 42.9% [7]. While no studies on delayed diagnosis of HD have used the Rintala score to assess bowel function, our comparison with non-delayed HD cases did not indicate a worse overall outcome. However, it is important to note that bowel function data were available for less than 50% of patients in both groups, limiting the strength of this analysis.

Unfortunately, we lack sufficient data to evaluate other long-term functional outcomes, such as sexual function, which has received considerable attention in recent literature [22]. Additionally, we cannot make inferences about mortality rates in the delayed-diagnosis group due to insufficient information on causes of death, nor can we assess mortality in undiagnosed HD patients who were never referred to our clinic.

Despite the limitations of a retrospective design, reliance on paper-based records, and challenges with patient follow-up common in LMIC settings, our study demonstrates the ability to collect detailed patient data and provide individualized care for HD patients. This highlights the potential for delivering high-quality outcomes even in resource-constrained environments. Efforts to overcome linguistic and cultural barriers, improve follow-up, and address systemic gaps remain vital to further enhancing care.

## Conclusions

The prevalence of late-diagnosed HD in our setting is similar to other LMICs. This is likely due to a combination of milder clinical presentations and delays in patient referral driven by socio-economic barriers.

Nevertheless, there is no observed difference between delayed and non-delayed groups in terms of procedures needed to manage HD, its complications, or bowel function outcomes. Moreover, our findings align closely with those reported in high-income countries.

To reduce delayed diagnosis, we advocate targeted education for peripheral hospitals, local clinics, and parents to improve awareness of colorectal conditions and warning signs.

## Data Availability

The datasets analysed during the current study are available from the corresponding author upon reasonable request.

## Abbreviations

HD: Hirschsprung Disease
RSB: Rectal suction biopsy
FTRB: Full thickness rectal biopsy
HAEC: Hirschsprung associated enterocolitis
LMIC: Low- and Middle-Income Country
